# Tumour Necrosis Factor-α Gene Polymorphism Is Associated with Metastasis in Patients with Triple Negative Breast Cancer

**DOI:** 10.1038/srep10244

**Published:** 2015-07-13

**Authors:** Hui-Hui Li, Hui Zhu, Li-Sheng Liu, Yong Huang, Jun Guo, Jie Li, Xin-Ping Sun, Chun-Xiao Chang, Zhe-Hai Wang, Kan Zhai

**Affiliations:** 1 Department of Medical Oncology, Shandong Cancer Hospital and Institute, Jinan, Shandong 250117, China; 2 Department of Radiation Oncology, Shandong Cancer Hospital and Institute, Jinan, Shandong 250117, China; 3 Department of Clinical Laboratory, Shandong Cancer Hospital and Institute, Jinan, Shandong 250117, China; 4 Department of Radiology, Shandong Cancer Hospital and Institute, Jinan, Shandong 250117, China; 5 Department of General Surgery, Beijing Chao-Yang Hospital, Capital Medical University, Beijing 100020, China; 6 Department of Clinical Laboratory, Shandong Provincial Hospital Affiliated to Shandong University, Jinan, Shandong 250117, China; 7 Medical Research Center, Beijing Chao-Yang Hospital, Capital Medical University, Beijing 100020, China

## Abstract

Tumour necrosis factor-α (TNF-α) is critical in the regulation of inflammation and tumour progression. TNF-α-308G > A is associated with constitutively elevated TNF-α expression. The purpose of this study was to assess the association between TNF-α-308G > A and breast cancer (BC) risk by subtype and the connection between genotypes and clinical features of BC. A total of 768 patients and 565 controls were enrolled in this study, and genotypes were detected using the TaqMan assay. No effect on susceptibility for any BC subtype was found for the TNF-α-308 polymorphism in our study or in the pooled meta-analysis. This polymorphism was shown to be associated with age at menarche in all BC and in progesterone receptor-negative BC. Interestingly, triple negative breast cancer (TNBC) patients with TNF-α-308A had an increased risk of distant tumour metastasis (OR = 3.80, 95% CI: 1.31–11.02, *P *= 0.009). Multi-regression analysis showed that TNF-α-308A was also a risk factor for distant tumour metastasis after adjustment for tumour size and lymph node metastasis status (OR* *= 6.26, 95% CI: 1.88–20.87, *P *= 0.003). These findings indicate that TNF-α might play a distinct role in the progression of TNBC, especially in distant tumour metastasis of TNBC.

Breast cancer (BC) is the most frequent type of malignancy in women in both the developed and the developing world^1^. It is a heterogeneous disease in regards to its clinical, histological and molecular profile. In recent decades, there has been great progress in the diagnosis of BC, but only oestrogen receptor (ER), progesterone receptor (PR) and human epidermal growth factor receptor2 (Her2) are typically used for BC diagnosis in routine clinical practice. BC intrinsic subtypes, including triple-negative breast cancer (TNBC) and Her2^+^, luminal A, and luminal B BCs, are characterized by immunohistochemistry (IHC) and have important differences in phenotype and prognosis^2^,^3^. Inflammation within the breast tumour microenvironment is known to be correlated with increased invasiveness and poor prognosis^4^. Pro-inflammatory biomarkers and the immune response are related to BC risk and/or prognosis^5^,^6^. However, many cytokines are involved in the pathogenesis of BC, and inflammatory processes and genes involved in cytokine-related functional pathways have gained increasing interest by researchers.

Tumour necrosis factor-α (TNF-α), a multi-functional cytokine, is involved in the promotion of inflammatory responses and plays a critical role in the pathogenesis of inflammatory, autoimmune and malignant diseases^7^. TNF-α is also a key molecule in the promotion of angiogenesis through the stimulation of endothelial cell proliferation and the enhancement of the expression of other pro-angiogenic factors^8^. Furthermore, TNF-α induces the expression of adhesion molecules involved in the invasion of metastatic tumour cells^9^,^10^. Elevated plasma levels of TNF-α have been detected in many malignancies and are often associated with poor prognoses^11^,^12^,^13^. Knockdown of the TNF-α gene is associated with cell proliferation inhibition and apoptosis in TNBC^14^.

Considerable evidence has shown that genetic variations, such as single nucleotide polymorphisms (SNP), in both tumour and host genomes have roles in the diagnosis, treatment outcome and survival of patients with cancer^15^,^16^. Increasing evidence has shown that a SNP in the promoter region of the TNF-α gene (−308G > A, rs1800629) causes genetic susceptibility to many types of tumours and autoimmune diseases, such as hepatocellular carcinoma, myeloma, lymphoma, ulcerative colitis, and Crohn’s disease^17^,^18^,^19^. The TNF-α-308A allele appears to have higher constitutive and inducible TNF-α expression, as the −308G > A mutation affects a consensus binding site of AP-2^20^,^21^. The association between the TNF-α-308 polymorphism and BC has been widely evaluated in different ethnicities; however, the results of these studies have been inconsistent, possibly due to BC heterogeneity and other factors^22^. It is unclear whether TNF-α-308G > A is associated with different BC subtypes and/or clinical features.

Because TNF-α is tumourigenic *in vitro* and *in vivo*, we hypothesized that the TNF-α-308G > A polymorphism may have an important function in different BC subtypes and be related to the characteristics of different BC subtypes, especially in highly aggressive BC. The current case-control study investigated the role of TNF-α-308G > A in BC susceptibility by IHC subtype and ER, PR and Her2 status and the relationship between genotypes and clinicopathological characteristics of BC.

## Results

Because the cases and controls were frequency-matched for age, there were no significant differences in the distributions of age between the cases and controls (*P *= 0.275). Of the 768 BC cases, 163 were TNBC and 82 were Her2^+^, 183 were luminal A, 340 were luminal B, 523 were ER^+^, 245 were ER^−^, 585 were PR^−^, and 686 were Her2^−^ BCs. The genotype frequency of TNF-α-308G > A in the controls was in concordance with Hardy-Weinberg equilibrium (HWE) (*P *= 0.940).

### TNF-α-308 polymorphism and BC

The allelic frequency of TNF-α-308A was 0.074 in the controls and 0.065 in all BC cases. Table 1 presents the distribution of genotypes for TNF-α-308G > A in the controls and BC cases. For all BCs, there was no association with the TNF-α-308 polymorphism (OR* *= 0.89, 95% CI: 0.63–1.25, *P *= 0.482) in the dominant genetic model. According to IHC classification, no association was found between the TNF-α-308 polymorphism and the TNBC (OR* *= 0.67, 95% CI: 0.38–1.19, *P *= 0.133), Her2^+^ (OR* *= 0.90, 95% CI: 0.41–1.72, *P *= 0.812), luminal A (OR* *= 0.67, 95% CI: 0.33–1.14, *P *= 0.120) or luminal B (OR* *= 1.20, 95% CI: 0.84–1.77, *P *= 0.523) subtypes. Consistent with the results for all BCs, no association was observed between the TNF-α-308 polymorphism and ER^+^ (OR* *= 0.98, 95% CI: 0.68–1.36, *P *= 0.852), ER^−^ (OR* *= 0.72, 95% CI: 0.43–1.18, *P *= 0.204), PR^−^ (OR* *= 0.92, 95% CI: 0.65–1.34, *P *= 0.867) or Her2^−^ (OR* *= 0.86, 95% CI: 0.63–1.35, *P *= 0.531) BCs.

Due to a lack of information, we conducted an updated meta-analysis to test whether the TNF-α-308 polymorphism is associated with overall BC risk rather than specifically testing its association with the various subtypes. Eighteen studies (including the present study) were included in this analysis, with a total of 13567 BC cases and 15087 controls. The detailed characteristics of each study in the pooled analysis are summarized in Table 2. Thirteen studies were conducted in Caucasians^23^,^24^,^25^,^26^,^27^,^28^,^29^,^30^,^31^,^32^,^33^,^34^,^35^, while the remaining 5 were conducted in Asians^36^,^37^,^38^,^39^. No association between the TNF-α-308 polymorphism and BC risk was found in Caucasians (OR* *= 1.06, 95% CI: 0.88–1.27, *P *= 0.555; I^2^* *= 80.7%, *P*_het_ < 0.001), Asians (OR = 1.07, 95% CI: 0.58–1.96, *P* = 0.826; I^2^ = 88.7%, *P*_het_ < 0.001) or all subjects (OR* *= 1.04, 95% CI: 0.87–1.24, *P *= 0.653; I^2^* *= 84.6%, *P*_het_ < 0.001) (Table 3). Begg’s funnel plot and Egger’s test were performed to evaluate the publication bias of all included studies. In the overall analysis, no evidence of obvious asymmetry for the TNF-α-308G > A polymorphism was found (*P*_Begg’s_ = 0.596). Additionally, the Egger’s test found no significant publication bias (*P* = 0.375).

### TNF-α-308 polymorphism and clinical features of BC

We analysed the association between TNF-α-308G > A and clinical characteristics (age at diagnosis, BMI, tumour stage, tumour size, lymph node metastasis, distant metastasis, age at menarche, menopause and family history of cancer) by BC subtypes classified using IHC (Table 4). In all BCs, the GA and AA genotypes were less frequent in patients who were 15 years old or more at menarche than inpatients who were under 15 years old at menarche (OR* *= 0.57, 95% CI: 0.35–0.95, *P *= 0.029). In the TNBC group, the GA and AA genotypes were associated with distant tumour metastasis (OR* *= 3.80, 95% CI: 1.31–11.02, *P *= 0.009). After adjusting for tumour size and lymph node metastasis status, this association remained (OR* *= 6.26, 95% CI: 1.88–20.87, *P *= 0.003), and in our study, this association was also statistically significant after adjusting for all clinical characteristics (OR = 5.83, 95% CI: 1.64–20.76, *P *= 0.004).

We analysed the association between TNF-α-308G > A and clinical characteristics in patients with different ER, PR, and Her2 statuses (Supplementary Table 1). A similar association between the GA and AA genotypes and age at menarche to that observed in all BCs was seen in PR^−^ patients (OR* *= 0.56, 95% CI: 0.32–0.98, *P *= 0.041).

## Discussion

Inflammatory cytokines play critical roles at different stages of tumour development and progression, including invasion and metastasis. Although several studies have reported an association between the TNF-α-308G > A polymorphism and BC risk, the results of those study were inconsistent. Moreover, little is known about this association in the Chinese. In the present study, we found that the TNF-α-308G > A polymorphism was not associated with the risk of BC in the different IHC subtypes or BCs classified based on ER, PR or Her2 status in Chinese, Asians or Caucasians. We provided evidence that the TNF-α-308 polymorphism is associated with age at menarche in all BCs and in PR^−^ BCs. Interestingly, patients with TNBC and the TNF-α-308A allele had an increased risk of distant tumour metastasis. Our study highlights the effect of the TNF-α gene polymorphism on the progression of BC by subtype. These findings suggest a potential connection between constitutively higher TNF-α expression and the pathogenesis of TNBC.

TNF-α is mainly produced by macrophages and is also expressed in a wide variety of cells, including mast cells, lymphoid cells, endothelial cells, cardiac myocytes, and fibroblasts^40^. Two TNF-α bioactive isoforms, a 26-kD transmembrane isoform and a 17-kD soluble isoform that under goes proteolytic cleavage by a metalloprotease, function by binding to TNF-α receptors (TNFRs)^41^. Two receptors (TNFR1 and TNFR2) bind TNF-α. TNFR1 is constitutively expressed in most tissues and can be activated by both transmembrane TNF-α (tmTNF-α) and soluble TNF-α (sTNF-α). When the death domain of TNFR1 interacts with the TNF-α receptor-associated death domain (TRADD), the resulting complex recruits proteins to activate apoptosis through caspase-3. TRADD can also bind TNF receptor-associated factor 2 (TRAF2) to recruit proteins that activate inhibitor of nuclear factor kappa-B kinase (IKK), germinal centre kinase (GCK), and receptor-interacting protein (RIP). These molecules then activate the nuclear factor kappa B (NF-κB), c-Jun N-terminal kinase (JNK), and mitogen-activated protein kinase (MAPK) pathways, which promote anti-apoptosis and cell survival. TNFR2 is generally expressed in cells of the immune system and can only bind sTNF-α. TNFR2 lacks a death domain and can also bind TRAF2 to activate an anti-apoptosis pathway^42^. Abnormal activation of JNK, MAPK, and NF-κB can lead to the aberrant expression of many genes that cause chronic inflammation, which stimulates tumour growth. Thus, tmTNF-α and sTNF-α act as immunoregulatory cytokines that connect inflammation with cancer progression.

Two recent meta-analyses reported that the TNF-α-308GA and AA genotypes were significantly associated with decreased BC risk in Caucasians^22^,^43^. However, the allele frequencies in the controls of some studies^44^,^45^ included in those two meta-analyses were not in accordance with HWE. Additionally, a meta-analysis by Yang *et al.* included one study that compared the frequencies of the different TNF-α-308 polymorphism genotypes in patients with benign breast disease and controls^46^ and another study that did not provide the frequencies of each genotype^47^. With strict inclusion criteria, we added new individual studies and performed an updated meta-analysis; for all BCs, we found no association with this polymorphism in Asians and Caucasians. It must be noted that BC is a complex disease with multiple environmental and genetic factors contributing to its progression. The lack of an association between TNF-α-308G > A and all BCs does not indicate that TNF-α-308G > A has no effect of susceptibility in certain subtypes. Future research is needed to clarify the connection between the higher constitutive TNF-α expression observed with the TNF-α-308G > A polymorphism and the risk of BC in each BC subtype.

TNBC is frequently observed in young patients and in patients with larger and higher-grade tumours^48^,^49^. TNBC is also associated with higher recurrence rates of metastasis and death, especially within 3 years of diagnosis^50^. TNBCs must have some specific and common pathways involved in metastasis. Our study provided some clarification of the distinct molecular pathway of distant metastasis in TNBC. It is known that TNF-α is involved in tumour metastasis through the stimulation of chemokines, which increases cell migration and invasion and promotes proliferation, and is involved in angiogenesis by increasing VEGF expression^51^,^52^. Our study suggests that higher constitutive TNF-α expression in patients with TNBC rather than other BC subtypes is associated with distant tumour metastasis. Previous studies also support our findings: knockdown of TNF-α gene expression through blockage of the NF-κB pathway inhibited cell proliferation and induced apoptosis in a TNBC cell line^14^; and in a murine model of TNBC, targeting TNF-related apoptosis-inducing ligand (TRAIL) receptor 2 suppressed TNBC tumour growth and metastasis^53^. Although sTNF-α originates from tmTNF-α, the function of these two isoforms are not exactly the same. Accumulating evidence shows that tmTNF-α might play an opposite role to that of sTNF-α. Tumour cells that express tmTNF-α are protected from apoptosis by the activation of NF-κB by sTNF-α through reverse signalling^54^. In tumour cells, the suppression of NF-κB reverse signalling by tmTNF-α resulted in higher cytotoxicity of sTNF-α^55^. We propose that higher constitutive TNF-α expression alters the ratio of tmTNF-α to sTNF-α and promotes TNBC cell growth. Future research should focus on how these two isoforms influence BC progression in various subtypes.

The major strengths of this study were the comprehensive analysis of theTNF-α-308 polymorphism in relation to susceptibility for various BC subtypes and its influence on the clinical features of BC, which will greatly help improve our understanding of the role of TNF-α in BC pathogenesis. The modest sample size of each subtype, which caused suboptimal statistical power, is the main limitation of this study; however, this could not be avoided. In conclusion, the present study shows that the TNF-α-308G > A polymorphism is not associated with BC risk but is associated with distant tumour metastasis in TNBC. This association might be mediated by the constitutively higher expression of tmTNF-α and/or sTNF-α in patients with the TNF-α-308A allele, promoting tumour growth through metastasis. Our results also confirm that targeting TNF-α suppresses TNBC progression.

## Patients and Methods

### Study subjects

This case-control study included 768 patients with constitutive BC and 565 cancer-free controls. All subjects were unrelated ethnic Han Chinese women. Patients were recruited from January 2010 to December 2013 at the Cancer Hospital, Shandong Academy of Medical Sciences and Beijing Chao-Yang Hospital, Capital Medical University and had been diagnosed with histologically confirmed BC. In this study, we classified the BC subtypes as TNBC (ER^−^, PR^−^ and Her2^−^), Her2^+^ (ER^−^, PR^−^ and Her2^+^), luminal A(ER^+^, PR^+^ and Her2^−^), and luminal B (ER^+^, PR^−^/PR_low_ and Her2^−^). The controls were randomly selected based on a physical examination in the same region during the same period as patient recruitment. The selection criteria included no history of cancer and frequency matching to cases by age. At recruitment, demographic information and clinical characteristics of each participant were collected. Informed consent was obtained from all participants. This study was conducted in accordance with the approved guidelines of the Institutional Review Board of the Cancer Hospital, Shandong Academy of Medical Sciences and Beijing Chao-Yang Hospital, Capital Medical University.

### TNF-α genotyping

Genomic DNA was extracted from peripheral blood lymphocytes of the study subjects. The genotypes of TNF-α at the -308 (G > A) site were analysed using a TaqMan genotyping platform (Roche LightCycler 480II, Roche Applied Science). The PCR primers were 5′-GGC CAC TGA CTG ATT TGT GTG T-3′ and 5′-CAA AAG AAA TGG AGG CAA TAG GTT-3′, and the probe sequences were 5′-FAM-CCC GTC CCC ATG CCC CTC-BHQ-3′ and 5′-TET-CTG AAC CCC GTC CTC ATG CCC-BHQ-3′. For quality control, genotyping was performed by researchers blinded to the case or control status, and a 10% random sample of cases and controls was genotyped twice by different persons; the reproducibility was 99.0%.

### Meta-analysis

We used three electronic databases (PubMed, Embase and Web of Knowledge) to identify relevant publications through December 2014 using key words related to the TNF-α gene polymorphism in combination with BC. The literature search was limited to English language publications on human studies. The reference lists of the relevant articles, reviews and editorials were also screened to find all additional eligible studies.

Studies were included in this meta-analysis if they met the following criteria: (1) case-control study design comparing TNF-α-308 polymorphism in BC cases and cancer-free controls; (2) cases were diagnosed histologically; (3)genomic DNA was isolated from peripheral blood leukocytes; (4) the study estimated the association between the TNF-α-308 polymorphism and BC risk; (5) sufficient data existed for calculating odds ratios (ORs) and corresponding 95% confidence intervals (CIs); and (6) the allele frequencies in the control group met HWE. If the same subject group was published in more than one study, the most complete study was selected for this pooled meta-analysis.

All information extracted from the included studies, including the first author, the year of publication, the ethnicity of the subjects, the number of subjects and genotype frequencies in the case and control groups, and the genotyping method, was double-checked and independently extracted from each publication by two investigators. Disagreements were resolved through discussion.

### Statistical analysis

χ^2^ tests were used to examine the deviation in genotype frequencies from HWE in controls and the differences in demographic variables and genotype distributions for different clinical features of BC. A dominant genetic model was used to estimate the associations between the TNF-α-308 polymorphism and the risk of BC by ORs and 95% CIs, which were calculated by unconditional logistic regression adjusted for age. For the meta-analysis, subgroups of studies based on ethnicity and experimental methods were determined as described in our previous study^56^. All statistical analyses were performed using Statistical Analysis System software version 9.2 (SAS Institute, Cary, NC,USA) and Stata 12.0 (StataCorp, College Station, TX, USA). A *P* value less than 0.05 was considered statistically significant.

## Additional Information

**How to cite this article**: Li, H.-H. *et al.* Tumour Necrosis Factor-α Gene Polymorphism Is Associated with Metastasis in Patients with Triple Negative Breast Cancer. *Sci. Rep.* **5**, 10244; doi: 10.1038/srep10244 (2015).

## Figures and Tables

**Table 1:** Genotype distribution of TNF-α-308G > A in controls and patients with BC.

Category	GG		GA + AA		OR (95% CI)	*P*
	n	(%)	n	(%)		
Controls (n* *= 565)	484	85.7	81	14.3	1.00	
All BC (n* *= 768)	668	87.0	100	13.0	0.89 (0.63–1.25)	0.482

IHC subtype						
TNBC (n* *= 163)	147	90.2	16	9.8	0.67 (0.38–1.19)	0.133
Her2^+^ (n* *= 82)	71	86.6	11	13.4	0.90 (0.41–1.72)	0.812
Luminal A (n* *= 183)	165	90.2	18	9.8	0.67 (0.33–1.14)	0.120
Luminal B (n* *= 340)	285	83.8	55	16.2	1.20 (0.84–1.77)	0.523

ER and PR status						
ER^+^ (n* *= 523)	450	86.1	73	13.9	0.98 (0.68–1.36)	0.852
ER^−^ (n* *= 245)	218	89.0	27	11.0	0.72 (0.43–1.18)	0.204
PR^−^ (n* *= 585)	503	86.0	82	14.0	0.92 (0.65–1.34)	0.867
Her2^+^ (n* *= 686)	597	87.0	89	13.0	0.86 (0.63–1.35)	0.531

Data were calculated by logistic regression adjusted for age with the TNF-α-308 variant genotype (GG) as the reference group.

**Table 2:** Characteristics of studies included in the pooled meta-analysis.

Ethnicity	Study	Samples (cases/controls)	Genotyping Method	Cases			Controls			*P*_HWE_ (controls)
				GG	GA	AA	GG	GA	AA	
Caucasian	Chouchane, 1997	40/106	ASPCR	15	24	1	72	33	1	0.183
	Mestiri, 2001	243/174	PCR-RFLP	167	53	23	117	53	4	0.480
	Giordani, 2003	125/100	PCR-RFLP	104	19	2	84	15	1	0.721
	Azmy, 2004	705/498	TaqMan	475	208	22	313	167	18	0.458
	Kamali-Sarvestani, 2005	223/235	ASO-PCR	192	31	0	203	32	0	0.263
	Scola, 2006	84/106	PCR-RFLP	71	12	1	79	26	1	0.472
	Gaudet, 2007	5156/4983	TaqMan	3679	1345	132	3488	1368	127	0.604
	Gonullu, 2007	38/24	PCRS-SPM	30	6	2	15	9	0	0.258
	Sirotkovic-Skerlev, 2007	158/76	TaqMan	136	22	0	68	8	0	0.628
	MARIE-GENICA, 2010	3138/5476	MALDI-TOF MS	2237	822	79	3795	1528	153	0.957
	Karakus, 2011	204/204	ASO-PCR	167	36	1	159	45	0	0.077
	Madeleine, 2011	869/900	SNPlex system	603	245	21	613	264	23	0.388
	Gómez Flores-Ramos, 2013	465/294	PCR-RFLP	302	96	67	254	38	2	0.660
Asian	Park, 2002	95/190	PCR-RFLP	75	20	0	134	54	2	0.174
	Kohaar, 2009	40/150	PCR-RFLP	21	16	3	211	24	2	0.170
	Pooja, 2011	200/200	Sequencing	179	19	2	166	34	0	0.189
	Xu, 2014	1016/806	PCR-RFLP	927	89	0	716	89	1	0.300
	This study	768/565	TaqMan	668	97	3	484	78	3	0.940

ASPCR: allelic specific polymerase chain reaction; ASO-PCR: allele specific oligonucleotide-polymerase chain reaction; MALDI-TOF MS: matrix-assisted laser desorption/ionization time-of flight mass spectrometry; PCR-RFLP: polymerase chain reaction-restriction fragment length polymorphism; PCRS-SPM:polymerase chain reaction sequence-specific primers method; SNPlex system is a genotyping platform of Applied Biosystem.

**Table 3:** Meta-analysis of the association between the TNF-α-308G > A polymorphism and BC in Caucasians and Asians (dominant model)

Ethnicity	Study (n)	Samples (cases/controls)	Cases			Controls			*P*_HWE_ (controls)	OR (95% CI)	*P*	I^2^ (%)	*P*_het_
			GG	GA	AA	GG	GA	AA					
Caucasian	13	11448/13176	8178	2919	351	9260	3586	330	0.437	1.06 (0.88–1.27)	0.555	80.7	<0.001
Asian	5	2119/1911	1870	241	8	1711	279	8	0.345	1.07 (0.58–1.96)	0.826	88.7	<0.001
Overall	18	13567/15087	10048	3160	359	10971	3865	338	0.912	1.04 (0.87–1.24)	0.653	82.7	<0.001

**Table 4:** Clinicopathological features of BC patients classified by IHC subtype and TNF-α-308 polymorphism.

Characteristics	All BC (n* *= 768)				TNBC (n* *= 163)				Her2^+^ (n* *= 82)			
	GG	GA+AA	OR (95% CI)	*P*	GG	GA+AA	OR (95% CI)	*P*	GG	GA+AA	OR (95% CI)	*P*
Age (years)												
≤45	369	43	1.00		49	8	1.00		18	5	1.00	
>45	399	57	0.89 (0.58–1.37)	0.604	98	8	0.50 (0.18–1.41)	0.184	53	6	0.41 (0.11–1.50)	0.167

Body mass index (kg/m^2^)												
≤25	353	54	1.00		81	13	1.00		38	5	1.00	
>25	321	37	0.75 (0.48–1.18)	0.211	72	4	0.35 (0.11–1.11)	0.064	33	6	1.38 (0.39–4.95)	0.618

Tumour stage												
I+II	375	47	1.00		80	7	1.00		31	4	1.00	
III+IV	393	44	1.20 (0.77–1.86)	0.419	67	10	1.71 (0.62–4.73)	0.300	40	7	1.36 (0.36–5.05)	0.649

Tumour size (cm)												
≤5	547	69	1.00		112	14	1.00		52	6	1.00	
>5	72	10	1.10 (0.54–2.23)	0.790	17	1	0.47 (0.06–3.81)	0.470	10	1	0.87 (0.09–8.00)	0.900

Lymph node metastasis												
No	275	35	1.00		70	6	1.00		29	5	1.00	
Yes	385	55	1.12 (0.72–1.76)	0.616	79	10	1.48 (0.51–4.27)	0.470	32	6	1.09 (0.30–3.95)	0.898

Distant metastasis												
No	617	74	1.00		125	10	**1.00**		66	11	1.00	
Yes	56	10	1.49 (0.73–3.04)	0.272	23	7	**3.80** (**1.31–11.02)**	**0.009**	5	0	NA	

Age at menarche (years)												
≤15	415	70	**1.00**		103	11	1.00		39	10	1.00	
>15	228	22	**0.57** (**0.35–0.95)**	**0.029**	47	5	1.00 (0.71–1.42)	0.995	29	3	0.40 (0.10–1.60)	0.186

Menopause												
Yes	423	54	1.00		72	8	1.00		37	5	1.00	
No	224	33	1.15 (0.73–1.83)	0.543	74	7	0.85 (0.29–2.47)	0.767	31	5	1.19 (0.32–4.51)	0.794

Family history of cancer												
No	531	75	1.00		118	11	1.00		53	10	1.00	
Yes	140	20	1.01 (0.60–1.71)	0.966	30	4	1.43 (0.43–4.81)	0.561	14	3	1.14 (0.28–4.69)	0.860

Characteristics	Luminal A (n = 183)				Luminal B (n = 340)			
	GG	GA+AA	OR (95% CI)	*P*	GG	GA+AA	OR (95% CI)	*P*
Age (years)								
≤45	73	8	1.00		129	22	1.00	
>45	92	10	0.99 (0.37–2.64)	0.987	156	33	1.24 (0.69–2.23)	0.472

Body mass index (kg/m^2^)								
≤25	90	12	1.00		144	24	1.00	
>25	75	6	0.60 (0.22–1.68)	0.326	141	21	0.89 (0.48–1.68)	0.726

Tumour stage								
I+II	104	11	1.00		160	25	1.00	
III+IV	61	7	1.09 (0.40–2.95)	0.873	125	20	1.02 (0.54–1.93)	0.941

Tumour size (cm)								
≤5	136	17	1.00		247	32	1.00	
>5	10	1	0.80 (0.10–6.64)	0.836	35	7	1.54 (0.63–3.76)	0.336

Lymph node metastasis								
No	77	7	1.00		99	17	1.00	
Yes	88	11	1.38 (0.51–3.72)	0.529	186	25	1.90 (0.79–4.55)	0.144

Distant metastasis								
No	153	17	1.00		273	36	1.00	
Yes	11	1	0.82 (0.10–6.73)	0.852	17	2	0.89 (0.20–4.02)	0.882

Age at menarche (years)								
≤15	110	14	1.00		163	35	1.00	
>15	53	4	0.59 (0.19–1.89)	0.372	99	10	0.52 (0.25–1.07)	0.070

Menopause								
Yes	99	11	1.00		215	30	1.00	
No	51	7	1.24 (0.45–3.38)	0.680	68	14	1.48 (0.74–2.94)	0.267

Family history of cancer								
No	117	15	1.00		243	39	1.00	
Yes	45	6	1.04 (0.38–2.85)	0.939	51	7	1.21 (0.51–2.84)	0.513

The total number of individuals may not be the same because of censored data.
